# How good is the orthopaedic literature?

**DOI:** 10.4103/0019-5413.40250

**Published:** 2008

**Authors:** Harman Chaudhry, Raman Mundi, Ishu Singh, Thomas A. Einhorn, Mohit Bhandari

**Affiliations:** Department of Surgery, McMaster University, Hamilton, Ontario, Canada; 1 Division of Orthopedic Surgery, Millcreek Community Hospital, Erie, PA, Canada; 2 Department of Orthopaedic Surgery, Boston University, Boston, MA, USA

**Keywords:** Critical appraisal, evidence-based medicine, quality, randomized trials

## Abstract

Randomized trials constitute approximately 3% of the orthopaedic literature Concerns regarding quality of the orthopaedic literature stem from a widespread notion that the overall quality of the surgical literature is in need of improvement. Limitations in surgical research arises primarily from two pervasive issues: 1) A reliance on low levels of evidence to advance surgical knowledge, and 2) Poor reporting quality among the high level surgical evidence that is available. The scarcity of randomized trials may be largely attributable to several unique challenges which make them difficult to conduct. We present characteristics of the orthopaedic literature and address the challenges of conducting randomized trials in surgery.

## EVIDENCE-BASED MEDICINE IN PRACTICE

In everyday clinical practice, evidence-based decision making depends on the integrated assessment of three critical factors-availability of clinical resources, patient values and the best-available evidence.^1^,^2^ As the need for orthopaedic care rises globally,^3^–^8^ it is imperative that these three factors guide orthopaedic care providers in their treatment decisions in order to ensure that patients receive optimal standards of care. Although judgments regarding clinical resources and patient values can be made on the physician's own accord, successful incorporation of evidence into the decision-making process depends directly on the existence of high quality literature. The following review will: 1) briefly outline the reporting practices that define high quality reporting and 2) assess the current quality of reporting in the orthopaedic literature.

## CHARACTERISTICS OF HIGH QUALITY EVIDENCE

### The hierarchy

With the numerous studies of varying credibility being published constantly, it is essential that physicians have a sound understanding of both study design and study quality. This will enable them to ‘critically appraise’ the studies they read and safely adopt changes to their practice.

Hierarchies of evidence have been established based on study design to aid physicians in evaluating the credibility of the studies they read. Different hierarchies of evidence exist for the various classes of studies, including those focusing on therapy, prognosis, harm, economic analysis and overviews. Studies focusing on therapy are the most common class of study found in the orthopaedic literature.^9^ A hierarchy of evidence for such studies is presented in Table 1. However, it must be noted that the quality of studies within each hierarchical category may vary based on the extent to which methodological safeguards are employed by study authors. As discussed below for randomized controlled trials, high quality reporting entails the adequate reporting of such methodological safeguards.

**Table 1:** Hierarchy of evidence for therapeutic decisions

	Study design	Description
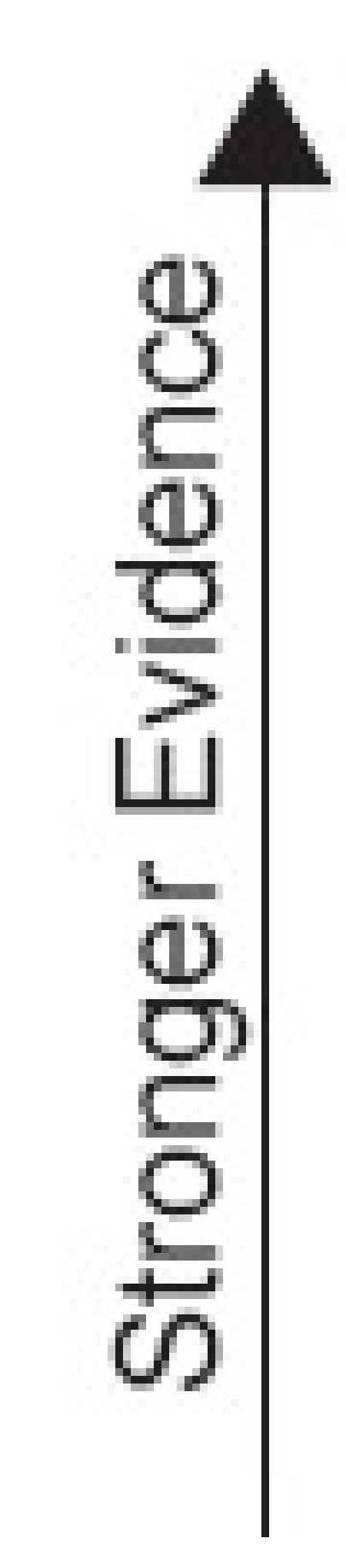	Systematic reviews of randomized trials	A systematic approach in which results of several RCTs are critically assessed and evaluated. Results may be pooled to arrive at single estimate of treatment effect
	Randomized controlled trial	A trial in which participants are randomly allocated to treatment or control interventions and prospectively followed to assess outcomes of interest
	Systematic reviews	A systematic approach in which results of several observational studies are critically assessed and evaluated. Results may be pooled to arrive at single estimate of treatment effect
	Observational studies	A study where physician or patient preference determines allocation to treatment or control intervention. Such studies do not employ the process of randomization
	Unsystematic clinical observations	A study in which a cohort of patients receive an intervention without a control group for comparison

Adapted from: Guyatt GH, Haynes RB, Jaeschke R, Cook D, Greenhalgh T, Meade M, et al.^2^

### Characteristics of high quality RCTs

The randomized controlled trial (RCT) has been established as the most secure study design for arriving at accurate estimates of treatment effects. This accuracy is contingent upon the process of randomization, which ensures that treatment and control groups are balanced for both known and unknown prognostic factors (characteristics of patients that influence outcomes). Although randomization can circumvent the introduction of bias by balancing groups for prognostic factors, there are several other measures-in addition to randomization-that must be taken to protect randomized trials from biased results.

One such measure referred to as allocation concealment entails that those recruiting patients into the study are unaware of which treatment arm the next patient will be delegated to. Failure to adequately implement allocation concealment in the design of an RCT has been shown to produce biased results that overestimate the effect of treatment.^10^ Other important safeguards that should be reported include blinding, loss to follow-up, sample size calculations and adherence to the intention-to-treat principle (i.e. analyzing patients in the groups they were originally randomized to regardless of which treatment they actually received).

Surgical randomized trials also have unique and special challenges that, if not addressed, can act as potential sources of bias. Firstly, surgical procedures consist of several components including preoperative care, the anesthetic procedure, the main intervention and post-operative care. Thus, only adequate reporting can allow readers to decipher which components of care were kept consistent or altered between the treatment arms. Secondly, in surgical procedures the skill and experience of the surgeon directly influences the patient's final outcome. Having treatment arms with imbalances in surgeon expertise would lead to the potential introduction of biased results. Finally, the type of setting (i.e. primary, secondary, tertiary or academic) and the volume of procedures performed at the study center prior to the study may also introduce bias if not balanced.^11^ Thus, adequate reporting of the treatment interventions, surgeon expertise and the study center are imperative.

### Characteristics of high quality systematic reviews [Table 1]

In contrast to unsystematic literature reviews, the systematic review attempts to collect, appraise, integrate and report results through a systematic process which limits bias.^12^ There are eight steps which are crucial to this process: 1) formulating a review question, 2) defining inclusion and exclusion criteria, 3) locating studies, 4) selecting studies, 5) assessing study quality, 6) extracting data, 7) analyzing and presenting results and 8) interpreting results.^13^ One of the most beneficial aspects of a systematic review is the meta-analysis. A meta-analysis is a quantitative analysis of results across many studies to arrive at the single best estimate of a treatment effect. The implications of the ability to provide a single best treatment effect while limiting bias is that the systematic review is a valuable tool for clinicians when making treatment decisions. Recognizing the potential of systematic reviews to positively impact clinical practice, the Quality of Reporting of Meta-Analyses (QUOROM) statement was developed to provide authors and readers with a general guide to methodological considerations in reporting and appraising a systematic review.^14^ In addition to familiarizing themselves with the QUOROM Statement, clinicians should ensure that systematic reviews are up-to-date, relevant (i.e. to the issue being considered), comprehensive (i.e. all relevant studies are included) and methodologically sound before implementing results into their clinical decision-making process.^13^,^15^

## WHY ARE WE CONCERNED? THE SURGICAL LITERATURE

### Overall quality of the surgical literature

Concerns regarding quality of the orthopaedic literature stem from a widespread notion that the overall quality of the surgical literature is in need of improvement. Limitations in surgical research arises primarily from two pervasive issues: 1) A reliance on low levels of evidence to advance surgical knowledge and 2) Poor reporting quality among the high level surgical evidence that is available.

The first issue was brought to the forefront of debate in a controversial paper published in the Lancet, criticizing the surgical literature for its reliance on case studies and case series-which constitute the lowest level of evidence available [Table 1]-as the primary source of evidence to advance surgical knowledge.^16^ Since then, researchers have largely supported the use of higher levels of evidence, noting that although case studies and case series do have an invaluable role in surgical research, these sources of evidence have limitations which simply prevent them from answering certain types of questions.^17^ For instance, Petrisor and Bhandari have outlined that these study designs are limited by their often retrospective nature, lack of a control group, incomplete data collection and generalizability.^9^ Therefore, as mentioned earlier, questions of effectiveness and efficacy are best answered using high quality RCTs and systematic reviews.

Fortunately, since criticism of the quality of surgical literature first surfaced, the number of published RCTs has grown rapidly.^18^ However, the publication of these high level trials is not sufficient. Recent evidence has criticized the reporting quality of available surgical RCTs, including those evaluating cardiothoracic, general, urological, gastrointestinal and vascular interventions.^19^–^21^ Jacquier and colleagues have made the pressing assertion that the quality of surgical RCTs in “in need of immediate improvement” after demonstrating that the reporting of methodological factors, treatment descriptions, care providers and treatment centers was poor across trials of various surgical subspecialities-30% of which were orthopaedic.^11^

The aforementioned evidence provides us with a brief overview which indicates that orthopaedic research is experiencing similar challenges and may need improvement. However, this evidence is marginal and cannot necessarily be extrapolated to the orthopaedic literature with certainty. Therefore, the remainder of this report will review the available evidence to determine whether the orthopaedic literature is experiencing similar quality ‘pitfalls’ and, if so, discuss the nature of these ‘pitfalls’.

## QUALITY OF THE ORTHOPAEDIC LITERATURE

### Randomized controlled trials

There is evidence that suggests the quality of reporting in orthopaedic RCTs needs of improvement.^18^,^19^,^22^–^25^ Several different quality checklists available to evaluate reporting-including the Detsky Quality Index, the CLEAR NPT and the CONSORT statement-have all been applied to the orthopaedic literature to demonstrate this poor state of affairs. One of the earlier studies to do so, carried out by Bhandari *et al.*, applied the Detsky scale to 72 randomized trials published in the Journal of Bone and Joint Surgery (American Volume) between 1988 to 2000. Briefly, the Detsky scale is comprised of fourteen items that assess reporting quality among five categories: 1) randomization, 2) outcome measures, 3) eligibility criteria and reasons for patient exclusion, 4) interventions and 5) statistical issues. A score of greater than 75% on this scale was deemed high quality. The study found that on average, only 68% of the Detsky items were reported, with 43 of the trials scoring below 75%.^18^ In addition to these findings, Bhandari and colleagues also highlighted some other interesting observations.

First and foremost, drug trials scored significantly better than surgical trials (73% vs. 64%, respectively) and an overwhelmingly greater number of drug trials were also reported as double-blinded compared to surgical trials (46% vs. 10%, respectively).^18^ This is particularly alarming, as the risk of introducing bias as a result of non-blinding is higher in non-pharmacological trials compared to pharmacological trials.^23^ This problem of inadequate blinding among nonpharmacolgical trials in the orthopaedic literature has been corroborated by a study that evaluated both non-pharmacological and pharmacological randomized trials on knee and hip osteoarthritis. The findings indicated that 65% of surgical trials that did not employ blinding could have feasibly done so.^23^

Secondly, Bhandari *et al.*, also demonstrated that cited statistical support and cited funding were correlated with higher quality reporting. Although funding may improve reporting practices, the source of funding always deserves a critical assessment, as industry-funded studies are significantly more likely to be “positive” studies with pro-industry outcomes. This holds true even when studies are controlled for their quality.^26^ Thus, it is imperative that trial authors 1) adequately report all methodological parameters utilized in their trials and 2) disclose the sources of funding, so that readers can sufficiently evaluate the validity of their trial results.

As the true clinical benefits of randomized trials can only be realized if manuscripts are thoroughly and accurately reported, several checklists have been developed to guide authors in writing their reports and in helping readers evaluate them. First published in the Journal of the American Medical Association in 1996, the Consolidated Standards of Reporting Trials (CONSORT) statement is considered as one of the more useful and popular of such guides. It is comprised of a comprehensive 22-item checklist and a flow diagram which collectively focus on the reporting of trial design, analysis, interpretation and participant progress.^27^ Several investigations applying the CONSORT statement to orthopaedic randomized trials have reinforced the poor quality of reporting.^21^,^24^,^25^ For instance, Bhandari *et al.*,^24^ carried out a comprehensive investigation in which they evaluated the extent to which reports of RCTs in orthopaedic trauma met the CONSORT criteria. Of the 196 reports (published across 32 journals) evaluated, it was found that the reports adhered to an average of only 32% ± 29% of the CONSORT criteria. Surprisingly, over 70% of the studies failed to meet even half of the criteria outlined in the CONSORT statement.^24^

To assist clinicians in evaluating scientific articles, several orthopaedic journals have implemented the Levels of Evidence rating system to their publication process, which pre-evaluates study reports and provides a quality rating for readers. Not only does this rating system enable readers to approach the “pre-appraised” trials with confidence, but it also allows journals to monitor the quality of the orthopaedic literature.^28^ In this rating system, clinical studies are rated on a scale of one to five (level one being the highest quality) based on quality and design. RCTs are designated a rating of level I or II, depending on the extent to which methodological safeguards are used to protect the study from bias. A recent evaluation of the orthopaedic literature found that only 11.3% of published papers are considered level I evidence according to this system.^29^ Even more alarming than the scarcity of level I evidence is that the reporting quality among level I studies has been called into question. In particular, Poolman and colleagues have found that level I studies published in the Journal of Bone and Joint Surgery (American Volume) have severe limitations in the reporting of methodological safeguards. They also found that the reporting quality in level I studies is not necessarily superior to level II studies.^28^

## SYSTEMATIC REVIEWS

The number of published systematic reviews in both the medical literature and the orthopaedic literature has grown rapidly over the past decade.^30^,^31^ Researchers and clinicians are increasingly coming to recognize the value of systematic reviews. For instance, a study of the orthopaedic literature by Bhandari and colleagues demonstrated that systematic reviews are more likely to be cited as evidence as compared to their unsystematic counterparts.^32^ Unfortunately, shortcomings continue to plague systematic reviews published in the orthopaedic literature and compromise the validity of their conclusions. An evaluation of the reporting quality of orthopaedic systematic reviews revealed that only 15% could be classified as methodologically rigorous.^31^ Systematic reviews that are identified as methodologically poor are also more likely to report positive outcomes when compared to high quality systematic reviews.^31^ Taken together, this suggests that nearly 85% of orthopaedic systematic reviews may assert biased conclusions.

One of the major shortcomings apparent in orthopaedic systematic reviews is the quality of the reviewed articles. The external validity of even a high quality systematic review is ultimately contingent on the quality of the included articles. Therefore, addressing the shortcomings of RCTs, as discussed in the previous section, is the first step to ensuring that systematic reviews produce the most valid and unbiased conclusions possible. Currently, however, the orthopaedic literature is experiencing a trend in which not only methodologically compromised RCTs but also *non-randomized* trials are being included in systematic reviews and meta-analyses. For instance, between 1996 and 2001, less than 15% of systematic reviews that analyzed only RCTs were published outside the Cochrane library. The majority of orthopaedic systematic reviews published in peer-reviewed journals included non-randomized trials.^33^ The most often cited reason for including non-randomized trials was lack of available RCTs in the orthopaedic literature. Perhaps most disconcerting is the fact that most systematic reviews and especially systematic reviews evaluating non-randomized trials, fail to assess the quality of included studies, thereby further compromising the validity of reported results.^33^

The orthopaedic literature has recently been scrutinized for the presence of publication bias. Publication bias occurs when positive trials are published more frequently than trials that demonstrate neutral results (i.e. those which did not find a statistically significant effect). This form of bias, also referred to as the ‘positive outcome bias’, is particularly elusive because it can corrupt even the most methodologically rigorous systematic reviews and bias results towards the direction of the positive effect.^34^ In a follow-up of 318 abstracts presented at the 1999 American Academy of Orthopaedic Surgeons annual meeting, Harris and colleagues demonstrated that trials with positive outcomes were far more likely to be published than trials with neutral outcomes.^35^ More recently, a landmark trial by Hasenboehler and colleagues reviewed 16,397 original surgical studies from 12 journals between 2000 and 2006, demonstrating that less than 10% were neutral studies whereas over 70% were positive studies.^36^ Subgroup analysis revealed identical trends of publication bias for orthopaedic journals. As systematic reviews are increasingly being referred to in clinical decision-making, this often unrecognized introduction of bias has been referred to as a “severe challenge to patient safety”.^36^

## FUTURE DIRECTIONS FOR IMPROVEMENT

Many of the issues discussed in this report can be addressed by conducting ‘higher level’ studies-in particular RCTs-and ensuring that trial manuscripts are reported thoroughly.

### Need for higher level orthopaedic evidence

Only 11.3% of studies published in the orthopaedic literature are considered level I. It has been further suggested that RCTs in particular constitute approximately 3% of the orthopaedic literature.^18^,^28^,^29^ To assume that orthopaedic researchers have not considered RCTs because of a lack of awareness is, perhaps, an overstatement. The scarcity of RCTs may be largely attributable to several unique challenges which make surgical RCTs difficult to conduct. We would like to address some of these challenges.

The first challenge has to do with inter-surgeon variability in skill and the associated ethical and practical concerns of randomization that this poses. From an ethical standpoint orthopaedic surgeons may not feel comfortable performing a surgical procedure which they are not particularly adept at. Some may feel it is unethical to provide a patient with a randomly assigned procedure which they do not feel is the ‘superior’ approach. From a practical standpoint, different surgeons will have different learning curves for a new procedure, which may serve as a source of bias. Devereux and colleagues have suggested the use of “expertise based” RCTs, in which patients are not randomized to a procedure *per se*, but rather to a surgeon with expertise in a particular procedure.^37^ It has been suggested that, among other things, expertise based RCTs will increase surgeon recruitment for trials, reduce bias associated with learning curves, prevent procedural crossovers and present a more ethical alternative to the traditional RCT.^37^

The second challenge involves the difficulty of blinding in a surgical trial. It has been suggested that lack of blinding in surgical trials may be attributable, at least in part, to a lack of awareness of available blinding methods.^38^ Researchers and readers must become thoroughly acquainted with various blinding methods. It should be noted that blinding methods may be categorized into three categories based on the group of individuals that may potentially be blinded: participants, health care providers and outcome assessors. There are extensive methods of blinding which further vary depending on the type of data being collected (i.e. participant-reported, physician-driven, paraclinical data or clinical event).^38^ Both researchers and readers are encouraged to become acquainted with the methods that Boutron and colleagues^38^ outline as a starting point for further investigation.

Another challenge involves the difficulty obtaining funding for surgical trials. Surgical researchers typically receive fewer grants, which are often of lesser value, than those received by their non-surgical counterparts.^39^ Surgeons need to work together and participate in clinical research, thus being better able to persuade funding sources as an organized lobby.^17^ Often surgeons will simply not conduct trials with new implants because of lenient regulatory practices and the ease with which implants can be introduced into clinical practice (as compared to pharmaceuticals).^39^ We hope that surgeons will recognize the value of RCTs in disseminating important innovations to their colleagues and, more importantly, to improving patient care and safety.

### Need for improved reporting of orthopaedic evidence

There is an immense need for improved reporting of orthopaedic RCTs. Chan and Bhandari have reinforced this notion through their recent assessment of 87 orthopaedic RCTs across eight medical journals.^40^ They found, as expected, reporting to be highly variable, with poor reporting for several methodological issues. However, after contacting the authors of these studies they concluded that “not reported” does not necessarily mean “not conducted”. The implication is that there is hope for a substantial improvement in the apparent quality of the orthopaedic literature through a mere improvement in reporting practices. Several checklists exist for various study designs that can improve reporting if adopted, endorsed and enforced by journals [Table 2]. We suggest that the submission of these quality checklists alongside manuscripts should become a strict part of the submission process. This would standardize reporting and enable clinicians to confidently interpret and incorporate study results into clinical practice.

**Table 2:** Checklists for improving the quality of reporting

Checklist	Evaluates (Study type)	Number of items
CONSORT	RCTs	22
CLEAR NPT	RCTs	15
QUOROM	Meta-Analyses of RCTs	18
STROBE	Observational Studies (Cohort, Case-Control and Cross-Sectional)	22
MOOSE	Meta-Analyses of Observational Studies	35

RCTs - Randomized controlled trials
